# Differences in airway lumen area between supine and upright computed tomography in patients with chronic obstructive pulmonary disease

**DOI:** 10.1186/s12931-021-01692-1

**Published:** 2021-03-31

**Authors:** Shotaro Chubachi, Yoshitake Yamada, Minoru Yamada, Yoichi Yokoyama, Akiko Tanabe, Shiho Matsuoka, Yuki Niijima, Wakako Yamasawa, Hidehiro Irie, Mitsuru Murata, Koichi Fukunaga, Masahiro Jinzaki

**Affiliations:** 1 Division of Pulmonary Medicine, Department of Medicine, Keio University School of Medicine, 35 Shinanomachi, Shinjuku-ku, Tokyo, 160-8582 Japan; 2 Department of Radiology, Keio University School of Medicine, 35 Shinanomachi, Shinjuku-ku, Tokyo, 160-8582 Japan; 3 Department of Clinical Laboratory, Keio University Hospital, 35 Shinanomachi, Shinjuku-ku, Tokyo, 160-8582 Japan; 4 Office of Radiation Technology, Keio University Hospital, 35 Shinanomachi, Shinjuku-ku, Tokyo, 160-8582 Japan; 5 Department of Laboratory Medicine, Keio University School of Medicine, 35 Shinanomachi, Shinjuku-ku, Tokyo, 160-8582 Japan

**Keywords:** COPD, CT, Pulmonary function test

## Abstract

**Background:**

No clinical studies to date have compared the inspiratory and expiratory airway lumen area between supine and standing positions. Thus, the aims of this study were twofold: (1) to compare inspiratory and expiratory airway lumen area (IAA and EAA, respectively) on computed tomography (CT) among supine and standing positions; and (2) to investigate if IAA and EAA are associated with lung function abnormality in patients with chronic obstructive pulmonary disease (COPD).

**Methods:**

Forty-eight patients with COPD underwent both low-dose conventional (supine position) and upright CT (standing position) during inspiration and expiration breath-holds and a pulmonary function test (PFT) on the same day. We measured the IAA and EAA in each position.

**Results:**

For the trachea to the third-generation bronchi, the IAA was significantly larger in the standing position than in the supine position (4.1–4.9% increase, all p < 0.05). The EAA of all bronchi was significantly larger in the standing position than in the supine position (9.7–62.5% increases, all p < 0.001). The correlation coefficients of IAA in the standing position and forced expiratory volume in 1 s were slightly higher than those in the supine position. The correlation coefficients of EAA or EAA/IAA in the standing position and residual volume, and the inspiratory capacity/total lung capacity ratio were higher than those in the supine position.

**Conclusions:**

Airway lumen areas were larger in the standing position than in the supine position. IAAs reflect airway obstruction, and EAAs reflect lung hyperinflation. Upright CT might reveal these abnormalities more precisely.

*Trial registration* University Hospital Medical Information Network (UMIN 000026587), Registered 17 March 2017. URL: https://upload.umin.ac.jp/cgi-open-bin/ctr/ctr_view.cgi?recptno=R000030456.

## Background

Chronic obstructive pulmonary disease (COPD) is a common, preventable, and treatable disease characterised by persistent respiratory symptoms and airflow limitation [1]. Airflow limitation in COPD is caused by decreased elastic recoil due to emphysematous destruction and small airways disease [2, 3]. Chest computed tomography (CT) can quantify these two main causes of COPD. Although small airways cannot be directly visualised by chest CT, a previous report has shown that CT measurements of central airway dimensions can estimate the dimensions of the histological small airways [4]. Early measurements of the airways have relied on manual tracing, but several automated methods have been developed. Previous reports using automated methods have shown that the mean lumen area of the 3rd to 6th generation airways reflects airflow limitation in patients with COPD [5, 6].

Objective measures of airway disease and emphysema have been well established using inspiratory CT [7]. Recently, central airway collapse using expiratory CT has been reported as an important clinical parameter in patients with COPD. Tracheal collapse during exhalation is prevalent in patients with COPD and related to worse St. George's Respiratory Questionnaire (SGRQ) scores and frequent exacerbation in these patients [8, 9]. However, only a few reports have shown that expiratory bronchial area and expiratory bronchial collapse can be CT biomarkers that reflect lung function abnormalities in patients with COPD [10, 11].

Lung hyperinflation is a crucial pathophysiological mechanism in the development of dyspnoea and exercise intolerance in patients with COPD [12]. Furthermore, it is a predictor of mortality in these patients [13]. Although lung hyperinflation is an important clinical indicator in patients with COPD, the relationship between the degree of lung hyperinflation and mean airway lumen area in inspiratory and expiratory CT has not been elucidated.

Recently, a 320-detector-row upright CT scanner has been developed to evaluate human anatomy in the upright position three-dimensionally, and clarify the effects of gravity on the human body [14]. In a previous report of healthy volunteers, we showed that the inspiratory and expiratory bilateral lung volumes were significantly higher in the standing position than in the supine position using an upright CT scanner [15, 16]. To the best of our knowledge, no clinical studies to date have accurately compared both the inspiratory and expiratory airway lumen areas (IAA and EAA, respectively) in the supine and standing positions. We hypothesised that the IAA, EAA and EAA/IAA ratio between supine and standing positions would be different, because the direction of gravity relative to the chest differs between the supine and upright positions. Thus, the aims of this study were: (1) to compare the airway lumen area on inspiratory and expiratory CT between supine and standing positions; and (2) to compare the relationship between airway lumen area on inspiratory and expiratory CT and lung function abnormalities such as airflow limitation and lung hyperinflation in patients with COPD.

## Methods

### Study population

This prospective study was approved by the Keio University School of Medicine Ethics Committee (No. 20160385). Written informed consent was obtained from all patients (UMIN Clinical Trials Registry [UMIN-CTR]: UMIN000026587). From August 2018 to September 2019, a total of 51 consecutive patients with known COPD, who were scheduled for clinical CT examination, were considered for inclusion in this prospective study. The exclusion criteria were as follows: age < 20 years old (n = 0); pregnant or unknown pregnancy status in patients of childbearing potential (n = 0); not able to undergo CT in a standing position (n = 0); not willing to provide written informed consent (n = 0); and forced expiratory volume in 1 s (FEV_1_)/forced vital capacity (FVC) > 70% on the day of the upright and conventional CT examinations (n = 3). Thus, 48 patients were included in this study.

### CT imaging protocol

**Fig. 1:**
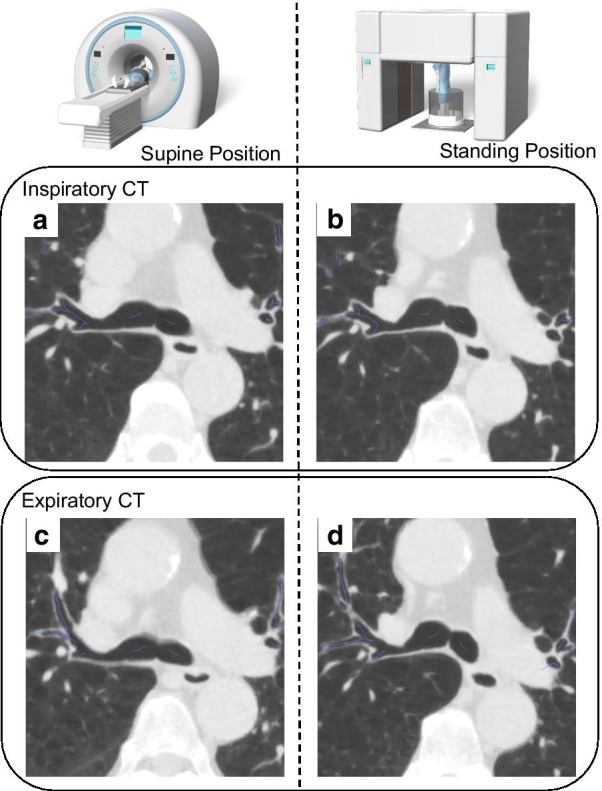
Axial CT images acquired at tracheal bifurcation level in a 74-year old male patient with COPD. **a** Inspiratory CT in the supine position. **b** Inspiratory CT in the standing position. **c** Expiratory CT in the supine position. **d** Expiratory CT in the standing position. The main bronchial areas were larger in the standing position than in the supine position on both inspiratory and expiratory CT. CT, computed tomography

All patients underwent conventional chest low-dose CT in the supine position with arms raised using a 320-detector-row CT (Aquilion ONE, Canon Medical Systems, Otawara, Japan), and upright chest low-dose CT in the standing position with arms down using a 320-detector-row upright CT (prototype TSX-401R, Canon Medical Systems) on the same day [14–17]. The chest scans in the two positions were unenhanced and were performed during both deep-inspiration and expiration breath-holds, with automatic exposure control using a noise index of 24 for a slice thickness of 5 mm (tube current range, 10–350 mA). The following scanning parameters were also the same for the supine and standing chest CT scans: peak tube voltage, 120 kVp; rotation speed, 0.5 s; slice collimation, 0.5 mm × 80; and pitch factor, 0.813. The series of contiguous 0.5-mm-thick images were reconstructed with Adaptive Iterative Dose Reduction 3D (Canon Medical Systems) [18]. The CT dose index volumes for the inspiratory supine CT, expiratory supine CT, inspiratory upright CT, and expiratory upright CT were 2.76 ± 0.53, 2.74 ± 0.54, 3.05 ± 0.61, and 3.36 ± 0.68 mGy, respectively; the dose-length products were 120.0 ± 27.0, 99.9 ± 19.0, 138.8 ± 28.2, and 127.0 ± 26.0 mGy-cm, respectively; the effective dose estimates were 1.68 ± 0.38, 1.40 ± 0.27, 1.94 ± 0.39, and 1.78 ± 0.36 mSv, respectively, and were determined by the dose-length product measurements and the appropriate normalised coefficient found in the literature for chest CT (0.014 mSv/(mGy-cm) [19]. Thus, the total effective dose estimate for the four CT examinations in this study was approximately 6.8 mSv, which is slightly less than that for a single routine clinical chest CT (7 mSv) [20]. Examples of low-dose conventional (supine position) and upright CT (standing position) images are shown in Fig. 1.

### Segmentation and measurement of the airway lumen

Airway segmentation and airway luminal area measurements were obtained using SYNAPSE VINCENT software (FUJIFILM Medical, Tokyo, Japan)^5^. The segmental bronchus was defined as the 3rd generation airway. Following automatic luminal segmentation of the airway tree, all branches of the 3rd to 6th generation airways in all segments were manually identified by tracking from the 3rd to the 6th generation [5, 21]. For each branch, cross-sectional images perpendicular to the longitudinal center line of the lumen were generated, and the lumen areas and internal diameters in the middle third portion were automatically measured and averaged. The mean lumen area for each generation airway was calculated in all segments.

### Pulmonary function test

All participants underwent a pulmonary function test (PFT) on the same day as they underwent both conventional and upright CT examinations (within two h of the CT examinations). The PFT was performed in a stable condition, with the patient in a sitting position, using a spirometer (Chestac-8900, Chest M.I., Tokyo, Japan) in accordance with ATS/European Respiratory Society recommendations [22, 23]. The residual volume and TLC were measured by the multi-breath helium dilution method. Predicted values of spirometric measurements were derived from the guidelines of the Japanese Respiratory Society [24, 25].

### Statistical analysis

Data are presented as means ± standard deviation (SD) or medians (interquartile range ([IQR]). Wilcoxon signed-rank test was performed to analyse differences in the airway lumen area between the supine and standing positions. The association between the airway lumen area in each position and parameters on PFT was evaluated by Spearman’s correlation test. The significance level for all tests was 5% (two-sided). All data were analysed using a commercially available software program (JMP version 14; SAS Institute Inc, Cary, NC, USA).

## Results

### Characteristics of the study population

The clinical characteristics of the study population are shown in Table 1. The median age of the patients with COPD was 77 years (IQR, 71–81); 91.7% were men, and 4.2% were current smokers. The number of patients with COPD among the Global Initiative for Chronic Obstructive Lung Disease (GOLD) grades 1, 2, 3, and 4 were 14, 27, 6, and 1, respectively.

**Table 1:** Baseline characteristics of the study population

	*N* = *48*
Sex, female, (%)	4 (8.3)
Age, years	77 (71–81)
Smoking index, pack-years	50.0 (30.0–76.9)
Current smokers, (%)	2.0 (4.2)
BMI, kg/m^2^	23.4 ± 3.2
FEV_1_/FVC, (%)	51.7 (43.9–61.4)
%FEV_1_, (%)	69.8 ± 19.1
COPD grades* 1/2/3/4, (%)	14/27/6/1 (29.2/56.3/12.5/2.0)

Data are presented as mean ± SD, medians (IQR), or number (%). * Defined by the Global Initiative for Chronic Obstructive Lung Disease. BMI, body mass index; FEV_1_, forced expiratory volume in 1 s; FVC, forced vital capacity; %FEV_1_, forced expiratory volume in 1 s as a percentage of predicted forced expiratory volume in 1 s; COPD, chronic obstructive pulmonary disease; GOLD, Global Initiative for Chronic Obstructive Lung Disease

### Comparison of the airway lumen area on inspiratory and expiratory CT between supine and standing positions

The evaluations of the airway lumen areas on inspiratory and expiratory CT scans are shown in Table 2. For the trachea to the 3rd generation bronchi, IAA was significantly larger in the standing position than in the supine position (trachea: 354.1, IQR 316.1–396.7 vs. 368.6, IQR 328.5–400.3, p = 0.0003; main bronchus: 195.4, IQR 176.2–229.2, vs. 204.9, IQR 188.4–231.7, p = 0.0098; 3rd generation bronchi: 115.3, IQR 97.9–141.0, vs. 120.5, IQR 98.9–147.4, p = 0.0359; supine vs. standing position, respectively).

**Table 2:** Comparison of airway lumen area at inspiratory and expiratory CT between the supine and standing positions

Generation	Supine	Standing	*p* value
IAA			
Trachea	354.1 (316.1–396.7)	368.6 (328.5–400.3)	0.0003
Main bronchus	195.4 (176.2–229.2)	204.9 (188.4–231.7)	0.0098
3rd	115.3 (97.9–141.0)	120.5 (98.9–147.4)	0.0359
4th	48.2 (38.1–58.6)	47.1 (36.4–61.4)	1.0000
5th	24.4 (18.2–27.8)	23.6 (17.1–31.2)	0.996
6th	11.7 (9.5–17.8)	11.6 (9.1–16.4)	0.1852
EAA			
Trachea	300.7 (269.4–332.9)	329.8 (294.4–355.7)	< 0.0001
Main bronchus	147.5 (91.6–175.1)	184.3 (160.8–206.9)	< 0.0001
3rd	69.8 (48.5–92.0)	82.6 (67.3–109.0)	< 0.0001
4th	23.5 (14.5–35.0)	30.0 (21.0–36.3)	0.0063
5th	9.7 (4.8–14.6)	12.6 (8.5–18.6)	0.0006
6th	3.2 (1.5–6.4)	5.2 (2.7–8.3)	0.0018
EAA/IAA			
Trachea	0.84 (0.76–0.90)	0.90 (0.84–0.95)	< 0.0001
Main bronchus	0.74 (0.50–0.85)	0.87 (0.80–1.00)	< 0.0001
3rd	0.65 (0.42–0.77)	0.78 (0.59–0.90)	< 0.0001
4th	0.52 (0.35–0.68)	0.62 (0.45–0.78)	0.0059
5th	0.40 (0.22–0.53)	0.53 (0.33–0.78)	0.0002
6th	0.29 (0.15–0.45)	0.45 (0.22–0.66)	0.0002

Data are presented as medians (IQR). IAA, inspiratory airway lumen area; EAA, expiratory airway lumen area; EAA/IAA; expiratory airway lumen area/inspiratory airway lumen area ratio

In contrast to the results of inspiratory CT, expiratory CT showed that the EAA of all bronchi was significantly larger in the standing position than in the supine position (trachea: 300.7, IQR 269.4–332.9, vs. 329.8, IQR 294.4–355.7, p < 0.0001; main bronchus: 147.5, IQR 91.6–175.1, vs. 184.3, IQR 160.8–206.9, p < 0.0001; 3rd generation bronchi: 69.8, IQR 48.5–92.0, vs. 82.6, IQR 67.3–109.0, p < 0.0001; 4th generation bronchi: 23.5, IQR, 14.5–35.0, vs. 30.0, IQR 21.0–36.3, p = 0.0063; 5th generation bronchi: 9.7, IQR 4.8–14.6, vs. 12.6, IQR 8.5–18.6, p = 0.0006; 6th generation bronchi: 3.2, IQR 1.5–6.4, vs. 5.2, IQR 2.7–8.3, p = 0.0018; supine vs. standing position, respectively).

On paired inspiratory and expiratory CT scans, the EAA/IAA of all bronchi was significantly larger in the standing position than in the supine position (trachea: 0.84, IQR 0.76–0.90, vs. 0.90, IQR 0.84–0.95, p < 0.0001; main bronchus: 0.74, IQR 0.50–0.85, vs. 0.87, IQR 0.80–1.00, p < 0.0001; 3rd generation bronchi: 0.65, IQR, 0.42–0.77, vs. 0.78, IQR 0.59–0.90, p < 0.0001; 4th generation bronchi: 0.52, IQR 0.35–0.68, vs. 0.62, IQR 0.45–0.78, p = 0.0059; 5th generation bronchi: 0.40, IQR 0.22–0.53, vs. 0.53, IQR 0.33–0.78, p = 0.0002; 6th generation bronchi: 0.29, IQR 0.15–0.45, vs. 0.45, IQR 0.22–0.66, p = 0.0002; supine vs. standing position, respectively). These results showed that bronchial lumen area expanded from the supine to the standing position, especially in expiratory CT.

### Correlations of airway lumen area at inspiratory CT in the supine and standing positions with the results of the PFT

The correlations between the IAA and the results of the PFT are shown in Table 3. For the 3rd to the 5th generation bronchi, IAA was significantly correlated with FEV_1_ and %FEV_1_ in the supine position (3rd generation bronchi: FEV_1_, ρ = 0.43; %FEV_1_, ρ = 0.44; 4th generation bronchi: FEV_1_, ρ = 0.38; %FEV_1_, ρ = 0.41; 5th generation bronchi: FEV_1_, ρ = 0.38; %FEV_1_, ρ = 0.34; all p < 0.05). For the main bronchus to the 6th generation bronchi, IAA was significantly correlated with FEV_1_ and %FEV_1_ in the standing position (main bronchus: FEV_1_, ρ = 0.32; %FEV_1_, ρ = 0.39; 3rd generation bronchi: FEV_1_, ρ = 0.50; %FEV_1_, ρ = 0.45; 4th generation bronchi: FEV_1_, ρ = 0.50; %FEV_1_, ρ = 0.41; 5th generation bronchi: FEV_1_, ρ = 0.43; %FEV_1_, ρ = 0.38; 6th generation bronchi: FEV_1_, ρ = 0.40; %FEV_1_, ρ = 0.29; all p < 0.05). RV, %RV and IC/TLC did not correlate with IAA both in the supine and standing positions, except for the 3rd generation bronchi with %RV (supine: ρ = − 0.39; standing: ρ = − 0.33). These results showed that the airway lumen area on inspiratory CT predicted airway obstruction but could not predict lung hyperinflation, and the correlation between IAA and the degree of airway obstruction was greater in the standing position than the supine position.

**Table 3:** Correlations of airway lumen area at inspiratory CT in the supine and standing positions with the results of the pulmonary function test

Generation	Supine	Standing	Generation	Supine	Standing
FEV_1_			RV		
Trachea	0.22	0.24	Trachea	0.10	0.06
Main bronchus	0.25	0.32**	Main bronchus	– 0.04	– 0.08
3rd	0.43**	0.50**	3rd	– 0.18	– 0.08
4th	0.38**	0.50**	4th	– 0.11	– 0.07
5th	0.38**	0.43**	5th	– 0.12	– 0.06
6th	0.32*	0.40**	6th	– 0.05	– 0.09
%FEV_1_			%RV		
Trachea	0.05	0.23	Trachea	– 0.13	– 0.16
Main bronchus	0.21	0.39**	Main bronchus	– 0.25	– 0.21
3rd	0.44**	0.45**	3rd	– 0.39*	– 0.33*
4th	0.41**	0.41**	4th	– 0.26	– 0.22
5th	0.34*	0.38**	5th	– 0.21	– 0.16
6th	0.21	0.29*	6th	– 0.12	– 0.13
			IC/TLC		
			Trachea	0.07	0.11
			Main bronchus	0.17	0.07
			3rd	0.26	0.20
			4th	0.12	0.08
			5th	– 0.01	– 0.06
			6th	– 0.05	– 0.03

*and ** indicate p < 0.05 and p < 0.01, respectively. FEV_1_, forced expiratory volume in 1 s; %FEV_1_, forced expiratory volume in 1 s as a percentage of predicted forced expiratory volume in 1 s; RV, residual volume; %RV, residual volume as a percentage of predicted residual volume; IC/TLC; inspiratory capacity/total lung capacity ratio

### Correlations of airway lumen area at expiratory CT in the supine and standing positions with the results of the PFT

The correlations between the EAA and the results of the PFT are shown in Table 4. For the main bronchus to the 4th generation bronchi, EAA was significantly correlated with FEV_1_ (main bronchus: ρ = 0.29; 3rd generation bronchi: ρ = 0.35; 4th generation bronchi: ρ = 0.29; all p < 0.05); however, EAA did not correlate with %FEV_1_ in the supine position. EAA in the supine and standing positions was significantly correlated with RV (main bronchus in the standing position: ρ = 0.31; 3rd generation bronchi: ρ = 0.37 vs. ρ = 0.37; 4th generation bronchi: ρ = 0.29 vs. ρ = 0.32; 5th generation bronchi: ρ = 0.28 vs. ρ = 0.30; 6th generation bronchi, ρ = 0.33 vs. ρ = 0.39; supine vs. standing position, respectively; all p < 0.05), and EAA in the standing position was significantly correlated with IC/TLC (5th generation bronchi: ρ = − 0.37; 6th generation bronchi: ρ = − 0.43; all p < 0.05). These results showed that the airway lumen area on expiratory CT predicts lung hyperinflation more precisely than airway obstruction, and the correlation between EAA and the degree of lung hyperinflation was slightly greater in the standing position than the supine position.

**Table 4:** Correlations of airway lumen area on expiratory CT in the supine and standing positions with the results of the pulmonary function test

Generation	Supine	Standing	Generation	Supine	Standing
FEV_1_			RV		
Trachea	0.24	0.26	Trachea	0.19	0.24
Main bronchus	0.29*	0.05	Main bronchus	0.31*	0.20
3rd	0.35*	0.31*	3rd	0.37**	0.37**
4th	0.29*	0.34*	4th	0.29*	0.32*
5th	0.28	0.19	5th	0.28*	0.30*
6th	0.24	0.15	6th	0.33*	0.39*
%FEV_1_			%RV		
Trachea	0.05	0.12	Trachea	– 0.01	– 0.06
Main bronchus	0.02	– 0.04	Main bronchus	0.04	0.10
3rd	0.11	0.07	3rd	0.07	0.12
4th	0.07	0.17	4th	0.08	0.12
5th	0.10	0.12	5th	0.11	0.21
6th	0.10	0.03	6th	0.19	0.31*
			IC/TLC		
			Trachea	0.02	0.02
			Main bronchus	– 0.02	– 0.08
			3rd	– 0.09	– 0.16
			4th	– 0.15	– 0.23
			5th	– 0.14	– 0.37*
			6th	– 0.22	– 0.43*

* and ** indicate p < 0.05 and p < 0.01, respectively. FEV_1_, forced expiratory volume in 1 s; %FEV_1_, forced expiratory volume in 1 s as a percentage of predicted forced expiratory volume in 1 s; RV, residual volume; %RV, residual volume as a percentage of predicted residual volume; IC/TLC; inspiratory capacity/total lung capacity ratio

### Correlations of EAA/IAA in the supine and standing positions with the results of the PFT

The correlations of EAA/IAA on paired inspiratory and expiratory CT scans with the results of the pulmonary function test are shown in Table 5. EAA/IAA in the supine and standing positions was significantly correlated with RV (main bronchus: ρ = 0.40 vs. ρ = 0.44; 3rd generation bronchi: ρ = 0.50 vs. ρ = 0.42; 4th generation bronchi: ρ = 0.36 vs. ρ = 0.30; 5th generation bronchi: ρ = 0.35 vs. ρ = 0.33; 6th generation bronchi: ρ = 0.37 vs. ρ = 0.44; supine vs. standing position, respectively; all p < 0.05) and %RV (main bronchus in the standing position: ρ = 0.34; 3rd generation bronchi: ρ = 0.33 vs. ρ = 0.40; 4th generation bronchi: ρ = 0.29 vs. ρ = 0.29; 5th generation bronchi in the standing position: ρ = 0.35; 6th generation bronchi: ρ = 0.29 vs. ρ = 0.39; supine vs. standing position, respectively; all p < 0.05). EAA/IAA in the supine position was not correlated with IC/TLC, but that in the standing position was significantly correlated with IC/TLC for the 3rd generation to the 6th generation bronchi (3rd generation bronchi: ρ = − 0.37; 4th generation bronchi: ρ = − 0.33; 5th generation bronchi: ρ = − 0.37; 6th generation bronchi: ρ = − 0.38; all p < 0.05). FEV_1_ and %FEV_1_ were not correlated with EAA/IAA both in the supine and standing positions, except for the main bronchus in the standing position, which was correlated with %FEV_1_ (ρ = − 0.39, p < 0.05). These results show that the ratio of the airway lumen area on inspiratory and expiratory CT scans predicts lung hyperinflation but does not predict airway obstruction, and the correlation between the EAA/IAA and lung hyperinflation was greater in the standing position than the supine position.

**Table 5:** Correlations of EAA/IAA in supine and standing positions with the results of the pulmonary function test

Generation	Supine	Standing	Generation	Supine	Standing
FEV_1_			RV		
Trachea	0.19	0.03	Trachea	0.11	0.28
Main bronchus	0.18	– 0.20	Main bronchus	0.40**	0.44**
3rd	0.15	– 0.13	3rd	0.50**	0.42**
4th	0.04	– 0.03	4th	0.36*	0.30*
5th	0.09	– 0.07	5th	0.35*	0.33*
6th	0.12	– 0.01	6th	0.37**	0.44**
%FEV_1_			%RV		
Trachea	0.11	– 0.10	Trachea	– 0.01	0.19
Main bronchus	– 0.08	– 0.39*	Main bronchus	0.15	0.34*
3rd	– 0.10	– 0.28	3rd	0.33*	0.40**
4th	0.15	– 0.15	4th	0.29*	0.29*
5th	– 0.06	– 0.12	5th	0.25	0.35*
6th	– 0.02	– 0.12	6th	0.29*	0.39**
			IC/TLC		
			Trachea	– 0.04	– 0.18
			Main bronchus	– 0.004	– 0.19
			3rd	– 0.26	– 0.37**
			4th	– 0.23	– 0.33**
			5th	– 0.16	– 0.37**
			6th	– 0.23	– 0.38**

^*^ and ** indicate p < 0.05 and p < 0.01, respectively. FEV_1_, forced expiratory volume in 1 s; %FEV_1_, forced expiratory volume in 1 s as a percentage of predicted forced expiratory volume in 1 s; RV, residual volume; %RV, residual volume as a percentage of predicted residual volume; IC/TLC; inspiratory capacity/total lung capacity ratio

## Discussion

**Fig. 2:**
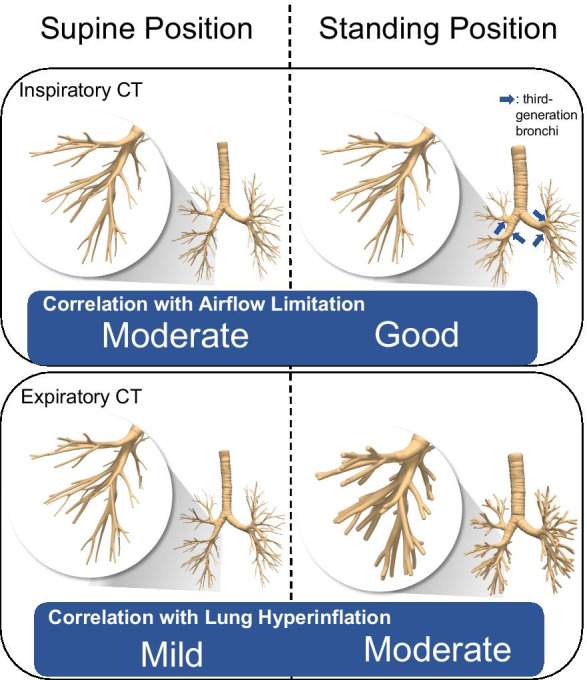
Difference of airway lumen area between supine and upright CT in patients with COPD. For the trachea to the 3rd-generation bronchi, the inspiratory airway lumen area was significantly larger in the standing position than in the supine position. Further, the expiratory airway lumen area was significantly larger in the standing position than in the supine position for all bronchi. Inspiratory airway lumen areas reflect airway obstruction and expiratory airway lumen areas reflect lung hyperinflation. Correlation coefficients among the airway lumen areas and lung function abnormalities were higher in the standing position compared to those in the supine position. CT, computed tomography; COPD, chronic obstructive pulmonary disease

To the best of our knowledge, this is the first study to show the difference in the airway lumen area between spine and standing position CT scans. The schema of this study is shown in Fig. 2. Our study provided three novel observations of potential relevance. First, the bronchial lumen area was larger in the standing position compared to that in the supine position on both inspiratory and expiratory CT. Second, IAA predicted airflow limitation, and both EAA and EAA/IAA predicted lung hyperinflation. Third, upright CT could predict airflow limitation and lung hyperinflation more precisely than conventional supine CT.

Our study showed that the bronchial lumen area, especially in the proximal airway, was larger in the standing position compared to that in the supine position. A possible reason for this was the difference of gravity on the bronchial lumen between the supine and standing positions. Changing gravity affects the upper airway area, lung volume and chest wall movement [26, 27]. It is possible that gravity directly changes the area of the trachea and bronchi, or indirectly changes the area of the airway, by changing abdominal and intrathoracic pressure via changing the movement of the tracheal smooth muscle, intercostal muscle and diaphragm. Recently, dynamic expiratory tracheal collapse has been reported as being a clinical indicator for predicting health status and future exacerbation in patients with COPD [9]. However, another report has shown that the magnitude of tracheal collapse is independent of disease severity and does not correlate significantly with physiologic parameters in patients with COPD [28]. This discrepancy might be caused by the difficulty in accurately measuring the magnitude of the collapse. The amount of collapse might be captured at arbitrary points in the respiratory cycle, potentially missing the inspiratory maximum and/or expiratory minimum [29]. Precise measurement of the dynamic expiratory tracheal collapse using upright CT might predict the clinical characteristics of patients with COPD. Thus, the clinical characteristics of having dynamic expiratory tracheal collapse in the standing position warrants further investigation.

In this study, the IAA for the 3rd to 5th generation bronchi was correlated with %FEV_1_ in the supine position, which was consistent with the results of previous reports using conventional supine CT [5, 6]. Further, the IAA for the main bronchus to the 6th generation bronchi was correlated with %FEV_1_ in the standing position. The correlation between the IAA and %FEV_1_ was stronger, and the IAA predicted %FEV_1_ in a wider range in the standing position compared to that in the supine position. A previous study reported that a change in the airway lumen area before and after bronchodilator treatment was a useful biomarker in patients with COPD [30]. Thus, accurate evaluation of changes to the airway lumen area using upright CT could be used to determine therapeutic effects in patients with COPD.

In the inspiratory CT, the IAA of the 6th generation bronchi was not correlated with %FEV_1_ in the supine position. Moreover, the correlation between the EAA and FEV_1_ was not greater in the 5th and 6th generation bronchi than that in the 4th generation. These results were not consistent with the previous reports showing that the IAA of 6th generation bronchi^6^ and EAA of 5th generation bronchi were more strongly correlated with %FEV_1_ and FEV_1_ [10]. This discrepancy could be explained by different sampling methods; for example, the previous studies only analysed selected airways, not all measurable airways. Further, the relatively small size of our study should also be noted.

Resting lung hyperinflation is a major manifestation of COPD. IC/TLC, which is known to be an indicator of resting hyperinflation, has been shown to predict mortality in patients with COPD [13], and deterioration of IC/TLC is associated with frequent exacerbations [31]. To the best of our knowledge, no study has reported an association between the spirometric indicator of lung hyperinflation and airway lumen area on the expiratory CT. The present study showed that the EAA and EAA/IAA can predict hyperinflation rather than airflow limitation. We emphasise that the EAA and IAA in the supine position did not correlate with IC/TLC, but the EAA and EAA/IAA of the distal bronchi were correlated with IC/TLC. Because of the complexity of the test, a lung volume measurement is not routinely recommended for patients with COPD. Recently, to reduce the spread of SARS-CoV-2, many pulmonary function testing laboratories have been closed or have significantly reduced their testing capacity [32]. Thus, the usefulness of upright CT evaluation of the airway lumen area for predicting lung hyperinflation might be clinically relevant for managing patients with COPD.

Some potential limitations of the present study should be discussed. First, the size of the study population was relatively small and had a relatively lower percentage of severe COPD patients. Second, the average age of the patients participating in our study was higher than that of other previous COPD clinical studies conducted in Western countries [33]. Further, the number of women was too low in our study. Previous reports have shown that airway wall thickness is related to sex, age, and smoking status [34]. Further studies with larger sample sizes, a representative percentage of severe COPD patients, and containing an appropriate number of women are needed. In addition, airway indices, such as the airway wall thickness at an internal perimeter of 10 mm, have been reported to be associated with subjective symptoms and quality-of-life scores in COPD patients [35, 36]. Further studies with several airway indices are needed for understanding the relationship between the airway indices of upright CT and clinical parameters.

## Conclusion

Upright CT quantification of the airway lumen area in patients with COPD revealed the difference in the airway lumen area between the supine and standing positions, and that the airway lumen areas on inspiratory and expiratory CT in the standing position are useful biomarkers for predicting airflow limitation and lung hyperinflation, respectively. Evaluation of airway lumen area using upright CT should be incorporated into a future COPD study.

## Data Availability

The datasets generated during and/or analysed during the current study are available from the corresponding author on reasonable request.

## Abbreviations

COPD: Chronic obstructive pulmonary disease
CT: Computed tomography
IAA: Inspiratory airway lumen areas
EAA: Expiratory airway lumen areas
PFT: Pulmonary function test
IQR: Interquartile range
FEV1: Forced expiratory volume in 1 s
%FEV1: Forced expiratory volume in 1 s as a percentage of predicted forced expiratory volume in 1 s
RV: Residual volume
%RV: Residual volume as a percentage of predicted residual volume
IC/TLC: Inspiratory capacity/total lung capacity ratio
